# Complex Tannins Isolated from Jelly Fig Achenes Affect Pectin Gelation through Non-Specific Inhibitory Effect on Pectin Methylesterase

**DOI:** 10.3390/molecules24081601

**Published:** 2019-04-23

**Authors:** Shang-Ta Wang, You-Jiang Feng, Ying-Jang Lai, Nan-Wei Su

**Affiliations:** 1 Department of Agricultural Chemistry, National Taiwan University, No. 1, Sec. 4, Roosevelt Road, Taipei 10617, Taiwan; d00623003@ntu.edu.tw (S.-T.W.); r96623009@ntu.edu.tw (Y.-J.F.); 2 Department of Food Science, National Quemoy University, No. 1, University Road, Jinning Township, Kinmen County 892, Taiwan; d91641006@gmail.com

**Keywords:** jelly fig achenes, pectin methylesterase inhibitor, bioassay-guided fractionation, antinutrients, tannins, proanthocyanidins

## Abstract

Jelly fig (*Ficus awkeotsang* Makino) is used to prepare drinks and desserts in Asia, owing to the gelling capability of its pectin via endogenous pectin methylesterase (PE) catalyzation. Meanwhile, substances with PE inhibitory activity (S_PEI_) in jelly fig achenes (JFA) residue were noticed to be able to impede the gelation. In this study, we characterized and isolated S_PEI_ from JFA by a series of PE inhibition-guided isolations. Crude aqueous extract of JFA residue was mixed with acetone, and 90% acetone-soluble matter was further fractionated by Diaion HP-20 chromatography. The retained fraction with dominant PE inhibitory activity was collected from 100% methanol eluate. Results from high-performance liquid chromatography mass spectrometry (HPLC/MS) and hydrolysis-induced chromogenic transition revealed the S_PEI_ as complex tannins. Total tannins content was determined in each isolated fraction, and was closely related to PE inhibitory activity. In addition, S_PEI_ in this study could inhibit activities of digestive enzymes in vitro and may, therefore, be assumed to act as non-specific protein binding agent.

## 1. Introduction

Pectin methylesterase (PE, EC 3.1.1.11) is a ubiquitous enzyme in plants, bacteria, and fungi [1,2]. PE catalyses the removal of methylester from homogalacturonan domains in pectin to release methanol and protons in the apoplast, which affects the properties of the pectin matrix by influencing calcium–pectate interactions [3]. Via demethoxylation process, pectin aggregates into calcium-linked gel structures, which increases wall porosity and reduces apoplastic pH, thought to activate local hydrolases including polygalacturonases and pectin lyases, thereby softening the cell wall and inducing the ripening of fruits [4].

In recent years, much research has been conducted to explore the substances with PE inhibitory activity (S_PEI_) which are substances capable of reducing the rate of de-esterification of PE. In the food industry, S_PEI_ were proposed to have several potential applications because of the strong interaction between inhibitory substances and PE. S_PEI_ may be used in fruit maceration and juice clarification processes by their controlled degradation of pectin [5]. Additionally, in wine production, S_PEI_ may reduce the methanol content by attenuating the activity of endogenous PE in fruit tissue [6]. Several inhibitory substances have been identified from plants: side-branched uronic acid in potato tubers; proteins (~16.3 kDa) in kiwifruit [7], *Arabidopsis* [8] and pepper leaves [9]; and catechins in green tea [10].

Jelly fig (*Ficus awkeotsang* Makino) is a native woody vine growing in Taiwan [11]. The seeds are used to make a jelly dessert, called “Ai-Yu-Tung,” in Asian countries [12]. For preparing the jelly dessert, the achenes from jelly fig (JFA) fruit are rubbed gently with the addition of hard water. The aqueous extract, which is rich in pectin, will then spontaneously form a pudding-like gel by demethoxylation of pectin catalyzed by endogenous PE. However, when achenes are crushed along with the process, the gelation ability as well as PE activity is eliminated. With this phenomenon, some S_PEI_ are assumed to exist in seeds to be released from the achenes. For elucidating the mechanism of PE activity removal, the identification of S_PEI_ in jelly fig achenes (JFA) is needed.

The aims of this study are to (i) isolate and characterize the JFA-S_PEI_ by performing a series of PE inhibition-guided purification and identification experiments including membrane separation, acetone precipitation, adsorbent precipitation, macroporous resin chromatography and acid hydrolysis, (ii) further reveal the composition of S_PEI_ by high-performance liquid chromatography-ultraviolet (HPLC-UV) and mass spectrometry (MS), and (iii) interpret the extensive enzyme inhibitory effect of JFA-S_PEI_ by conducting trypsin and α-amylase inhibition experiments. In addition, since the JFA-S_PEI_ were identified to consist predominantly of complex tannins in our study, total tannins content were determined within each isolated fraction as well.

## 2. Results

### 2.1. Effect of Molecular-Weight Fractionation and Polyvinylpolypyrrolidone (PVPP)/Protein Treatments on Inhibition Efficacy of Jelly Fig Achenes (JFA-S_PEI_)

**Figure 1:**
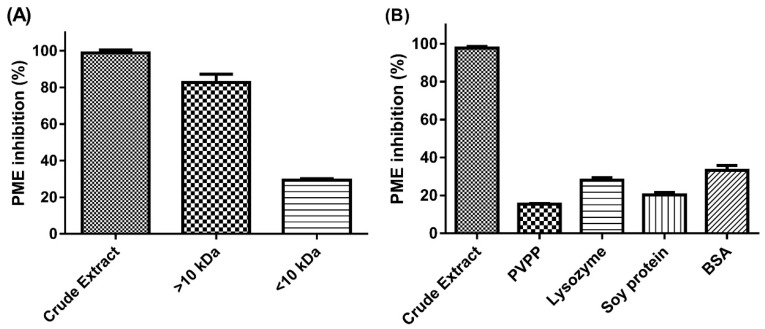
Relative inhibition of pectin methylesterase (PE) activity (%) by (**A**) jelly fig achenes (JFA) S_PEI_ crude extract and its membrane separation fractions (MW cutoff [MWCO], 10 kDa), and (**B**) crude extract after precipitation by polyvinylpolypyrrolidone (PVPP) and proteins. Data are mean ± standard deviation (SD), *n* = 3. BSA, bovine serum albumin. S_PEI_, substances with pectin methylesterase inhibitory activity.

Crude extract powder of JFA was first dissolved in NaCl solution, then boiled and centrifuged to eliminate PE and pectin residues. Molecular-weight fractions of the supernatant were obtained by membrane separation (MWCO, 10 kDa). The crude extract showed significant PE inhibition (98.9%), and inhibitory potency was almost 1.5 times higher with the >10-kDa than <10-kDa MWCO fraction (82.7% and 29.4%, respectively) (Figure 1A). The inhibitory activity was mostly from the >10-kDa fraction.

At first, we considered S_PEI_ in JFA as proteinaceous molecules. Therefore, we used polyvinylpolypyrrolidone (PVPP) and protein interaction assessment. Insoluble PVPP can be used to specifically remove compounds with phenolic group such as proteins and tannins from solution via hydrogen binding [13]. With supernatant from crude extract treated with PVPP subjected to PE inhibition assay, the inhibitory capacity was reduced to 15.3% as compared with while the control group was 97.7% (Figure 1B). These results indicate that JFA-S_PEI_ possesses remarkable capacity to bind with PVPP to form a precipitated complex. For further identification, the crude solution incubated with proteins (soy protein, lysozyme and BSA) underwent PE inhibition test as well. The solution became turbid and was precipitated during this process. PE inhibition was significantly reduced to approximately 20% to 30% (28.0%, 20.3% and 33.3% with lysozyme, soy protein and BSA, respectively) (Figure 1B). The binding capacities of JFA-S_PEI_ toward different proteins are significant as well. Therefore, phenolic compounds are supposed to be mostly responsible for the reduced PE activity in crude extract and fractions.

### 2.2. Fractionation of Crude JFA-S_PEI_ by Acetone Precipitation 

**Figure 2:**
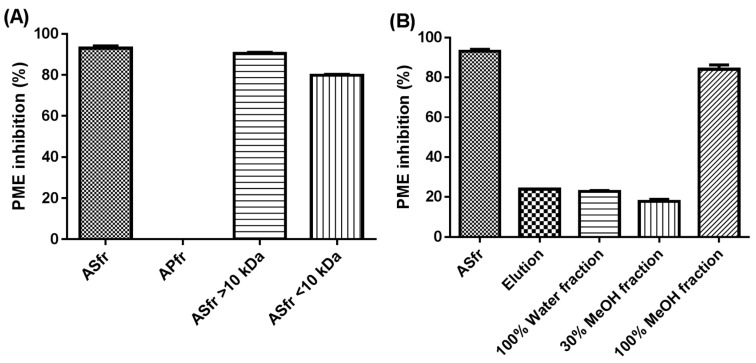
Relative inhibition of pectin methylesterase (PE) activity (%) by (**A**) acetone-soluble of S_PEI_ (ASfr) and precipitated fractions (APfr) and ASfr’s membrane fractions (MWCO, 10 kDa), and (**B**) eluted fractions of ASfr (0%, 30% and 100% methanol) by Diaion HP-20 column chromatography. Data are mean ± SD, *n* = 3. The PE inhibition of APfr was not detectable in triplicate tests. S_PEI_, substance with pectin methylesterase inhibitory activity.

To confirm whether the inhibitory substances are belonging to proteinaceous substances or not, the crude extract was subsequently fractionated by acetone precipitation, commonly used to precipitate and concentrate proteins, to isolate the proteinaceous substances. The acetone precipitant was collected and the solvent was evaporated under a gentle stream of nitrogen, then reconstituted with 1.5% NaCl solution. Consequently, PE inhibition of the precipitant was not observable (0%) (Figure 2A), so the proteinaceous fraction could not perform PE inhibitory activity, whereas the acetone-soluble fraction (ASfr) showed 93.1% inhibitory activity. According to the inhibitory activity of the ASfr, protein may not be considered in the active composition of JFA-PE inhibitory effect. Furthermore, with both ultrafiltration fractions (MWCO, 10 kDa) of ASfr, inhibition was comparable to that with the ASfr (90.5% and 79.9%, respectively) (Figure 2A). Hence, nearly all of the S_PEI_ were localized in the acetone-water soluble fraction, which may contain a correspondingly high level of phenolic compounds.

### 2.3. Further Fractionation of S_PEI_ by HP-20 Macroporous Resin Chromatography

**Figure 3:**
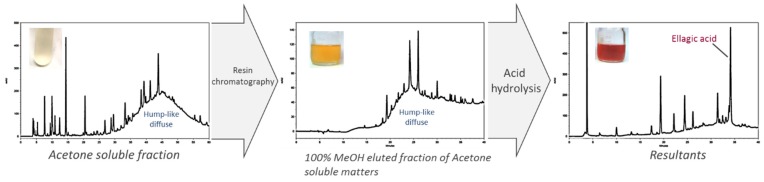
Changes in chromatographic profiles and appearance of S_PEI_ acetone-soluble fraction (ASfr), 100% methanol fraction of ASfr, and its acid hydrolysis resultant (1 N HCl, 121 °C for 30 min). S_PEI_, substances with pectin methylesterase inhibitory activity.

With this experiment, we aimed to establish whether the S_PEI_ in the (90:10) acetone-water soluble fraction could be further purified by using macroporous resin adsorption chromatography with a Diaion HP-20 column. The ASfr was further fractionated in distilled water and 30% and 100% methanol. The water effluent and 30% methanol effluent both showed approximately 20% inhibitory capacity (Figure 2B) so most inhibitory substances were adsorbed on the resin under these elution conditions and were desorbed and eluted out by 100% methanol because this effluent exhibited 84.2% inhibition of PE. Comparison of ASfr and the 100% MeOH fraction (100% MeOHfr) in HPLC chromatograms (Figure 3) showed a removal effect of related polar compounds in the ASfr fraction by the retention behaviour of those that were non-polar, and this effluent fraction still had a comparable inhibitory activity. Therefore, Diaion HP-20 chromatography can be used as an effective purification procedure for JFA-S_PEI_ identification.

### 2.4. High-Performance Liquid Chromatography (HPLC) Analysis of Acid Hydrolysate of S_PEI_ Fractions

Figure 3 shows the HPLC chromatogram of the ASfr at ultraviolet (UV) 267 nm; a hump diffuse peak profile appeared at RT = 44.5 min. This broad hump eluted over a 30-min period and was thought to represent the elution of tannins [14,15]. Although most condensed tannins are eluted by HPLC as a single, broad peak (or hump), additional tannins also elutes throughout the chromatogram as a broad bleed, causing a raised baseline. We used acid hydrolysis followed by membrane dialysis to determine the characteristics of S_PEI_. The hump diffuse peak profile disappeared after acid hydrolysis of the ASfr in the HPLC chromatogram (Figure S1), and PE inhibitory ability was diminished (~30%, data not shown). In addition, a chromogenic transition from pale yellow to light pink was observed within acid hydrolysis process, indicated the presence of polymeric phenolic components. These results strongly support that proanthocyanidin compounds were responsible for the PE inhibition in JFA residue. Furthermore, the inhibitory substances were hypothesized to be polymeric phenols such as proanthocyanidins and ellagitannins according to the characteristics of the tannic compound that appeared in the hydrolysis procedure.

### 2.5. Characterization of 100% MeOHfr by Ninhydrin Test

To determine whether polypeptides are involved in the PE inhibition in JFA, we used ninhydrin test with the 100% MeOHfr and gelatin solution before and after hydrolysis according to the standard procedure and measured the absorbance at 570 nm. The absorbance of gelatin (Table 1) was elevated (>25 times) after acid hydrolysis according to the formation of a colored ninhydrin complex with free amino groups released from the polypeptide backbone of gelatin. Nevertheless, the 100% MeOHfr remaining at a low level of absorption indicated that the content of this fraction should not be polypeptides.

### 2.6. Identification of JFA-S_PEI_ by HPLC-Ultraviolet (UV) and Mass Spectrometry (MS) Spectrum

With the aforementioned results, JFA-S_PEI_ was suggested to involve tannins. Thus we used enzymatic and acid hydrolysis assay followed by reverse-phase HPLC analysis to further establish whether the 100% MeOHfr could be tannic compounds and predict the classification of this tannin. Commercial tannase was incubated with the MeOHfr for a catalyzed hydrolysis reaction acting on carboxylic ester bonds between digallate structures in gallotannins. As compared with no tannase treatment, the tannase treatment conferred no significant release of tannin subunit from the fraction (data not shown). In contrast, acid hydrolysis resulted in release of ellagic acid (Figure 3), the main derivative of ellagitannins by spontaneous lactonization after hydrolysis, eluted at retention time of 34.2 min. No gallic acid peak was identified in chromatograms. Additionally, acid hydrolysis conferred a color transition from yellow-brown to red, a typical characteristic of tannins (Figure 3). The results provide direct evidence that the 100% MeOHfr may be composed of several types of tannins including ellagitannins but not gallotannins.

**Figure 4:**
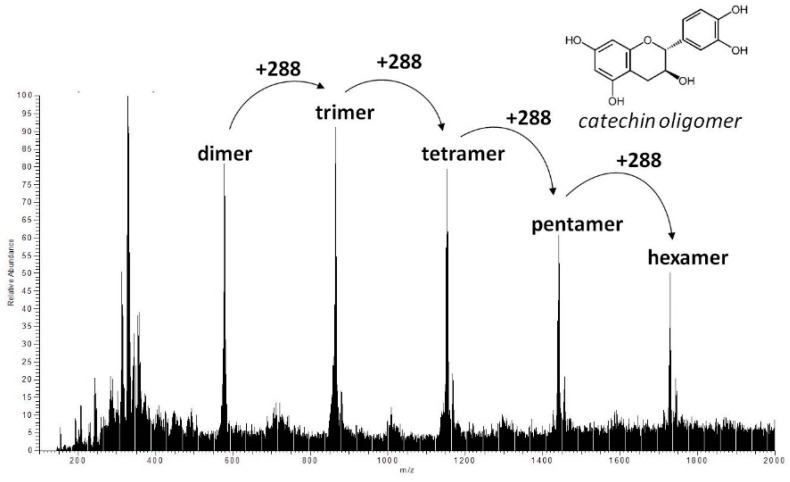
Mass spectrometry (MS) spectra for 100% methanol fraction of ASfr. Procyanidin oligomer and catechin monomer were identified under fragmentation of tested substances. ASfr, 90% acetone soluble fraction of S_PEI_ from jelly fig achenes. S_PEI_, substances with pectin methylesterase inhibitory activity.

Furthermore, the 100% MeOHfr was introduced into electrospray ionization/mass spectrometry (ESI/MS) analysis in negative ion mode with continuous-flow injection because the tannin molecules are better detected this way than with liquid chromatography owing to the large number of isomers, which results in an unresolved hump at the end of chromatograms and lead to low sensitivity of tannic compounds [16]. Figure 4 shows molecular ion species consistent with procyanidin oligomers containing singly linked units. A series of abundant ions separated by 288 Da was observed from *m*/*z* 577 to 1729, corresponding to the molecular masses of procyanidins with DP 2–6. Indeed, these signals could be interpreted as [M − H]^−^ ion peaks of dimeric (*m*/*z* 577), trimeric (*m*/*z* 865), tetrameric (*m*/*z* 1153), pentameric (*m*/*z* 1441), and hexameric (*m*/*z* 1729) procyanidins, respectively. Hence, 100% MeOHfr was composed of ellagitannins and also proanthocyanidins.

### 2.7. Inhibition of Digestive Enzymes

We hypothesized that the high protein-binding capacity of tannic compounds leads to an interaction between S_PEI_ and digestive enzymes, and subsequently diminishes the catalytic activities of these enzymes on the substrates. Thus, we assessed the hydrolysis activity of α-amylase and trypsin pre-treated with ASfr and the 100% MeOHfr (100 times diluted). α-Amylase inhibitory activity was observed in the ASfr and the 100% MeOHfr, both fraction inhibited the activity of pancreatic α-amylase (76.8% and 78.4% with ASfr and the 100% MeOHfr, respectively) In addition, pretreatment with S_PEI_ fractions greatly decreased the hydrolysis activity of trypsin on casein (88.6% and 70.2% with ASfr and the 100% MeOHfr, respectively) (Table 2). Accordingly, JFA-S_PEI_ may reduce the activity by forming a precipitated complex with trypsin.

### 2.8. Total Tannins Contents

The tannin levels of each isolated fraction were addressed in Table 3. Approximately 1.7 mg of tannins could be extracted from a gram of dry JFA with crude extraction process. Acetone soluble fraction retained almost 80% tannin from crude extract, and possessed significant PE inhibitory capability. Along with HP-20 chromatography, tannins were most preserved in the 100% MeOHfr, which conferred high inhibitory effect toward PE. In addition, acid hydrolysis processes seriously diminished the contents of tannins from 1.41 to 0.06 mg/g DW for ASfr and from 1.35 to 0.04 mg/g DW for 100% MeOHfr. Results from quantitation of tannins showed high consistency with the PE inhibitory activity of each fraction.

## 3. Discussion

Recent evidence demonstrated that S_PEI_ has an important physiological role in PE regulation in plants because of an ability to inhibit plant PE activity [17,18,19]. Therefore, S_PEI_ has several potential applications in a food-technological context [20]. These endogenous substances found in several plant species such as kiwi fruit, *Arabidopsis*, pepper and broccoli, were identified as proteinaceous compounds [7,8,9,17]. Active S_PEI_ were found in JFA and characterized as thermotolerable polypeptides of 3.5 to 4.5 kDa by Jiang et al. [11,21]. However, in our study, several findings indicated that JFA-S_PEI_ mostly consisted of endogenous ellagitannins and proanthocyanidins. Jiang et al. revealed that JFA-S_PEI_ extracts had remarkable inhibitory effect on PE activity after a 30-min boiling treatment and were considered thermotolerable substances [11], and they identified JFA-S_PEI_ extracts by membrane separation followed by Sepharose G-50 chromatography, the S_PEI_ showed competitive inhibition with substrates [21]. However, by following these procedures, we found several polymeric phenolic characteristics such as color transition after acid hydrolysis and a hump diffuse peak profile in chromatograms of isolated fractions with PE inhibitory activity. Therefore, we aimed to characterize the composition of JFA-S_PEI_ by using a series of PE inhibition-guided purification experiments.

S_PEI_ in JFA showed high thermostability because of remarkable inhibition of PE activity after the heating process described previously. Jiang, Li, Chang, and Chang, (2002) reported that the 3~10-kDa MWCO fraction of JFA-S_PEI_ crude extracts conferred greater inhibition of PE activity than other fractions [21], but we found inhibitory substances in the >10-kDa MWCO fraction. Furthermore, with acetone precipitation, PE inhibition was performed with both the >10-kDa and <10-kDa MWCO fraction of the acetone-soluble part. This finding is probably due to the synthesis of the non-covalent interaction between S_PEI_ and protein that formed a complex structure and was retained in the high MW fraction, then the addition of acetone broke the binding and led to substance release to the low MW fraction. Hence, acetone precipitation could be considered a purification process of JFA.

A negative result from the ninhydrin test clearly revealed that in the presence of JFA-S_PEI_, very few or no free amino groups remained after hydrolysis. Moreover, the results of PVPP and protein interaction assessment were direct evidence that JFA-S_PEI_ had protein-binding capacity and was implicated with phenolic compounds. Crude extract solution showed clear-turbid transition and significantly reduced PE inhibition after PVPP/proteins were introduced. This is an indication of complex formation and was probably due to S_PEI_ mostly consisting of phenolic compounds.

In addition, Jiang, Li, Chang, and Chang, (2002) reported that incubation of JFA-S_PEI_ with trypsin reduced PE inhibition to ~3%, which suggests that its cleavage ability toward polypeptide chain contributed to the inhibition of PE activity [21]. However, we found evidences that reduced PE activity may involve tannic compounds but not protein. Pre-treatment of trypsin with S_PEI_ fractions reduced the hydrolysis activity of trypsin toward model protein, which provide strong support for our hypothesis that the reduced PE inhibitory activity of JFA-S_PEI_ involved a precipitated complex formed between S_PEI_ and trypsin but not trypsin-catalyzed hydrolysis.

Several phenolic compounds have been found to possess inhibitory effect of PE activity. Lewis et al. (2008) found that green tea catechins can be used to inhibit PE activity across plant taxa and that the inhibitory interaction occurred at the substrate binding site of PE [10]. Chen et al. (2009) showed that the addition of gallic acid and coumaric acid in the wine-making process may reduce methanol content by a mixed inhibition pattern of PE activity [22]. Previous studies indicated that phenolic compounds might have potential inhibitory activity toward PE.

We found a hump diffuse peak profile in HPLC chromatogram of both JFA-S_PEI_ fractions that disappeared after acid hydrolysis, followed by downregulation of PE inhibition. This finding may be due to tannin content responsible for the reduced PE activity. Moreover, ellagic acid was observed after hydrolysis, but no gallic acid was released during this process. However, MS spectra showed molecular ion species consistent with procyanidin oligomers containing singly linked units. Therefore, we concluded that the purified JFA-S_PEI_ was a mixture of ellagitannins and condensed tannin, and these tannic compounds may play a more important role than the proteinaceous inhibitor found in JFA.

The term “tannin” was suggested to be reserved for phenolic compounds with a sufficient degree of hydroxylation and molecular size to form complexes with proteins and other polymers under suitable conditions of pH and concentration [23]. Low MW phenolics including phenolic acids and simple flavonoids may bind protein but cannot crosslink the complexes, as is required for precipitation [24]. Our results are consistent with these findings. The precipitated complexes we observed after the addition of soy protein, lysozyme and BSA may be due to the formation of ellagitannin/condensed tannin–protein complexes.

Tannins are widely considered to reduce activities of enzymes via non-specific protein binding. The inhibition activities of trypsin, α-amylase and lipase were found positively and linearly related to the degree of polymerization of tannins [25]. As well, condensed tannins inhibited activities of lactate dehydrogenase, alcohol dehydrogenase and α-glucosidase [26]. In our study, JFA-S_PEI_ with tannic substances inhibited digestive enzyme including α-amylase and trypsin, and may further be regarded as non-specific inhibitory substance of PE. On the other hand, hydrolysis of dietary polysaccharide such as starch is the dominant source of blood glucose that causes hyperglycemia [26]. This reaction is carried out by a group of hydrolysis enzymes that includes pancreatic α-amylase. It is believed that inhibition of these enzymes can be a crucial strategy for management of type 2 diabetes [27]. Therefore, JFA-S_PEI_ may lead to potential diabetes prevention effects.

Total tannin content of each fraction was determined along with the isolation process. We found relatively high tannin contents in fractions that conferred high PE inhibitory activities. This result provides firm evidence for the fact that JFA-S_PEI_ consists predominantly of tannic substances. In phytophysiology, endogenous tannins of plants were reported to affect the physiological condition of plants. For example, gallo- and ellagitannins were found to powerfully inactivate the early stages of pectolysis of low-methoxy pectin catalyzed by pectinase [28]. Furthermore, to our knowledge, our study is the first to demonstrate that tannins inhibit the activity of plant source PE. The results suggest that these endogenous polyphenol compounds via inhibiting PE may regulate the physiologic development of some tannin-rich plant organs.

## 4. Materials and Methods

### 4.1. Materials and Chemicals

Jelly fig (*Ficus awkeotsang* Makino) and, pea (*Pisum sativum* L.) for PE extraction, were purchased from a local market in Taipei, Taiwan. Soy protein was from Gemfont (Taipei). Methylene blue and sodium chloride were from Riedel-de Haën (Seelze, Germany). Acetone, methanol, hydrochloride solution and fluorescein isothiocyanate (FITC)-casein were from Merck (Darmstadt, Germany). Trypsin solution was from ThermoFisher Scientific (Waltham, MA, USA). Pancreatic α-amylase was from Megazyme (Bray, Ireland). Methyl red was from Ferak (Berlin, Germany). Polyvinylpolypyrrolidone (PVPP), ninhydrin, pectin, lipase, tannase, tannic acid, Folin–Ciocalteu reagent, sodium carbonate, dinitrosalicylic acid, bovine serum albumin (BSA) were from Sigma Aldrich (St. Louis, MO, USA). The DIAION^®^ HP-20 macroporous resin was from Mitsubishi Chemical (Tokyo, Japan). All chemicals were of reagent grade.

### 4.2. Preparation of JFA-S_PEI_ Crude Extract and Pectin Methylesterase (PE) Solution

Preparation of S_PEI_ crude extract from JFA was as described previously [21]. In brief, JFA was first washed with water flowing continually in a cheesecloth bag to remove pectin and to obtain JFA residue. An amount of 50 g dried JFA residue was introduced into 500 mL distilled water and gently stirred overnight at room temperature to eliminate the remaining pectin. JFA residue suspension was homogenized with a polytron homogenizer (Kinematica AG Littau, Switzerland) at 2000 rpm for 20 min to extract S_PEI_, the extraction solution was centrifuged at 3000× *g* for 10 min and supernatant was collected. The residual precipitate was then extracted twice by the same procedure; the supernatants were combined and heated at 95 °C for 15 min to deactivate the PE, then the solution was diluted with 3% NaCl solution to a volume of 1 L and boiled to eliminate residual PE. Resulted solution was centrifuged at 6500× *g* for 20 min to obtain the supernatant as a S_PEI_ crude extract.

Extracting PE from pea-pod shells was as described previously [11]. Briefly, 500 g pea-pod shells were homogenized for 2 min in 2 L cold (4 °C) distilled water. The mixture was filtered through cheesecloth to obtain the residues. Pea-pod residues were then extracted with a two-fold (*v*/*w*) volume of 0.3 M NaCl solution; the extract was filtered and the filtrate as PE solution was stored at 4 °C for further use.

### 4.3. Fractionation of S_PEI_ Crude Extract

Preliminary fractionation involved acetone precipitation. Ice-cold acetone at 90% (*v*/*v*) was gently added with constant stirring. The mixture was incubated overnight at −20 °C or below. The solution was then filtered with filter paper and the soluble part was concentrated by rotary evaporation. The ASfr was obtained by reconstitution to the original volume with distilled water. The solid content was dried at room temperature and considered the acetone-precipitated fraction of S_PEI_ crude extract (APfr).

For further fractionation, an amount of 60 mL ASfr underwent Diaion HP-20 chromatography with a water–methanol gradient system to obtain a water-eluted fraction and 30% and 100% methanol-eluted fraction (60 mL for each fraction).

Membrane separation (MW cutoff [MWCO] 10 kDa) involved using an Amicon ultraflitration cell equipped with an ultrafiltration membrane (Millipore) to obtain <10 and >10 kDa fractions from S_PEI_ crude extract.

### 4.4. Evaluation of PE Inhibitory Activity

**Figure 5:**
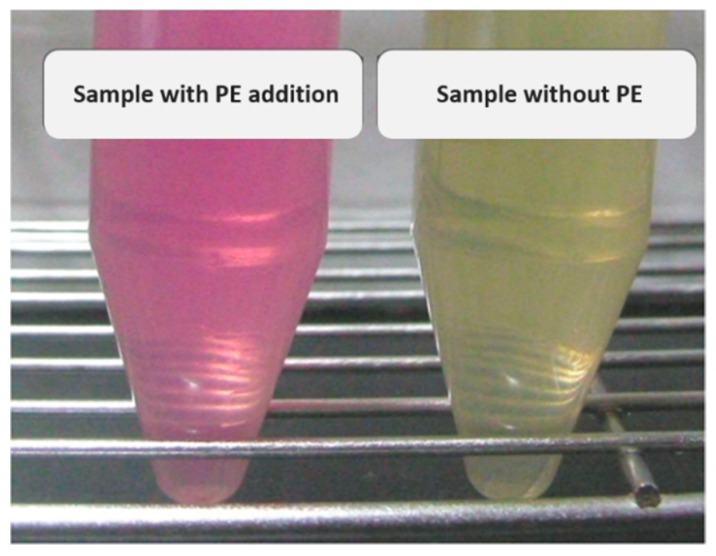
Chromogenic transition of pectin methylester (PE) removing reaction with the methyl red-methylene blue indicator. With PE addition, the solution turned red (left) while the blank solution remained light yellow (right).

To evaluate the inhibitory activity of S_PEI_, 200 μL PE solution was mixed with 800 μL S_PEI_ solution at various concentrations and incubated at 30 °C for 10 min, then 5 mL of 0.5% pectin solution (dissolved in 0.1 M NaCl) was added into the reaction mixture and incubated at 40 °C for 1 h. At the end of incubation, the mixture was heated at 95 °C for 15 min to terminate the enzyme catalysis reaction. PE inhibitory activity was evaluated by using methyl red-methylene blue indicator. Through this indicator, the cleavage of methylester groups and release of hydrogen ions could be detected by monitoring the pH-induced chromogenic transition with visual observation (Figure 5). An amount of 20 mg methyl red powder was dissolved in 60 mL ethanol and diluted with distilled water to a volume of 100 mL; methylene blue was dissolved in 100 mL distilled water to a concentration of 20 ppm. The indicator solution was obtained by mixing these two solutions. Eventually, 100 μL of methyl red-methylene blue indicator was introduced into the solution and PE activity was estimated by measuring absorbance at 527 nm, the values of which showed linear changes along with increasing concentration of hydrogen ions. PE inhibition was calculated as percentage of control. The blanked absorbance of pectin solution with the addition of PE and without adding S_PEI_ was considered as 100%.

### 4.5. Chemical Characterization of S_PEI_

To characterize JFA-S_PEI_, we used several assessments and treatments, including PVPP/protein treatment, ninhydrin test, acid hydrolysis and trypsin inhibition test. To explore the effect of interaction between S_PEI_ and PVPP on PE inhibitory activity, 200 mg PVPP was introduced into 10 mL S_PEI_ crude extract and stirred at room temperature for 1 h, then centrifuged at 3000× *g* for 10 min [29]. The supernatant was diluted to the original volume with distilled water, and PE inhibitory activity of S_PEI_ was performed. Proteins including soybean protein, lysozyme and BSA were added to S_PEI_ crude extract to determine the effect on PE inhibitory activity of S_PEI_. Protein solutions at 1% (*w*/*v*) were mixed with the same volume of S_PEI_ crude extract solution and incubated at 4 °C overnight, then underwent the PE inhibition analysis. PE inhibition was also calculated.

To further identify the composition of JFA-S_PEI_, we used acid hydrolysis by incubation with 1 N HCl at 121 °C for 30 min. The pH value was adjusted to 6 with approximately 200 μL of 40% NaOH. The hydrolyzed ASfr fraction was then analysed by high-performance liquid chromatography (HPLC) to detect hydrolysis products.

The ninhydrin test was performed as described [30]. The 100% MeOHfr from HP-20 chromatography was dried by rotating evaporation, and 0.1 g of the dried substances underwent acid hydrolysis as described previously. After cooling to room temperature, the pH was adjusted to 5 with 1 M NaOH. The sample was then dehydrated, and dried materials were reconstituted with 50% ethanol and introduced into 0.2% ninhydrin solution. The reaction was allowed to proceed for 15 min in a boiling water bath. After cooling to room temperature, the sample was diluted with 50% ethanol and the absorbance at 570 was measured. Hydrolyzed gelatin was used as a polypeptide control.

### 4.6. HPLC and MS Analysis

HPLC involved use of an analytical-model 584 solvent delivery gradient HPLC module (ESA Biosciences, Chelmsford, MA, USA), a Rheodyne injector model 7725i with a 20 μL sample loop (Rheodyne, Cotati, CA, USA), a 250 × 4.6 mm column, 5-μm Water Polarity TMdC18 protected by a guard cartridge (Hichrom 5C18, Berkshire, UK) and a UV detector model UV6000LP (Thermo Separation Products, San Jose, CA, USA). Chromatographic separation was performed at 25 °C by use of a linear mobile phase gradient including 0.1% acetic acid in water (solvent A) and 0.1% acetic acid in methanol (solvent B) at flow rate 0.65 mL/min. Elution involved a gradient starting with 1% B in A to reach 50% B at 60 min and then returning to the initial conditions for 10 min (1% B in A). Samples were recorded at 267 nm. For analysing the composition of 100% MeOHfr, elution involved a gradient starting with 1% B in A to reach 60% B at 30 min, 80% B at 40 min, and 90% at 50 min and then returning to the initial conditions for 10 min at a flow rate of 0.75 mL/min. Samples were recorded at 255 nm and 267 nm. Gallic acid and ellagic acid were identified according to their retention times by chromatographic comparisons with authentic standards. MS detection of the 100% MeOHfr was conducted with an electrospray source for the Finnigan LCQ advantage electrospray ion-trap mass spectrometer (Thermal; San Jose, CA, USA) at a constant flow rate of 10 μL/min by using a model 22 medical syringe infusion pump (Harvard Apparatus, South Natick, MA, USA) with a 100-μL syringe. The MS spectrum was performed with a sheath gas flow rate of 50 arb, an AuxSweep gas flow rate of 10 arb, a spray voltage of −3.5 kV and a capillary temperature of 225 °C.

### 4.7. Digestive Enzyme Inhibition Assay

A trypsin inhibition test was used to explore whether S_PEI_ could affect the activity of trypsin on a model protein. ASfr and the 100% MeOHfr were diluted 100 times with distilled water and mixed with 0.5 μg/mL trypsin at 1:19 volume. The mixture was incubated at 28 °C for 10 min, then a 100 μL aliquot was mixed with 100 μL of 0.5 μg/mL FITC-casein solution. The reaction was allowed to proceed at 28 °C for 20 min. Fluorescence emission was obtained with excitation 485 nm and emission 538 nm. A calibration curve was produced with FITC-casein from 10 to 500 ng/mL.

For the α-amylase inhibition, a mixture of 80 μL ASfr or the 100% MeOHfr and 20 μL 0.9% sodium chloride solution containing α-amylase (13 U/mL) were incubated at 37 °C for 10 min. After preincubation, 50 μL 1% soluble starch in 0.9% sodium chloride solution were added to each tube at timed intervals. The reaction mixtures were then incubated at 37 °C for 10 min followed by the addition of 100 μL dinitrosalicylic acid color reagent. The test tubes were then placed in a boiling water bath for 5 min to stop the reaction and cooled to room temperature. The reaction mixture was then diluted with 1 mL distilled water and absorbance was read at 540 nm. The digestive enzymes inhibition was calculated as percentage of control. The blanked absorbance of reaction solution with the addition of digestive enzymes and without adding S_PEI_ was considered as 100%.

### 4.8. Determination of Tannins Level

Total tannins contents were determined as described [31] with modification. Firstly, total phenol content of sample was assessed. For the assay, 400 μL of diluted sample were added to 2 mL of 1:10 diluted Folin–Ciocalteu reagent. After 4 min, 1.6 mL of saturated sodium carbonate solution (75 g/L) was added. After 2 h of incubation at room temperature, the absorbance at 760 nm was measured in triplicate. Tannic acid (0–3 μg/mL) was used for calibration of standard curve. The results were expressed as milligram tannic acid equivalent (mg TAE)/g dry weight of JFA.

For determining total tannins content, subsequently, 1 g of casein was added into 18 mL of the diluted sample. The mixture was kept under mechanical agitation for 3 h at room temperature, and then centrifuged at 3000 rpm for 20 min. The supernatant was collected and assessed for total phenolic content by method mentioned above. The tannins level is calculated as the difference between the total phenol level and the non-complex residual phenol level, once the tannins are removed from the medium through complexation with casein.

### 4.9. Statistical Analysis

One-way analysis of variance (ANOVA) with Duncan’s multiple comparison was used to determine statistical significance. Differences were considered statistically significant at *P* < 0.05.

## 5. Conclusions

In conclusion, we isolated S_PEI_ from JFA and characterized them to be complex tannins consisting of ellagitannins and condensed tannins. Acetone precipitation and Diaion HP-20 chromatography could be effective methods for purifying JFA-S_PEI_. Purified S_PEI_ could inhibit digestive enzymes via non-specific inhibitory effect and are potent antinutrients. This study could improve our understanding of the characteristics of S_PEI_ in JFA, and the approaches could be useful for isolating these endogenous inhibitory substances.

## Figures and Tables

**Table 1:** Chromogenic capabilities of gelatin and methanol fraction of S_PEI_ from jelly fig (*Ficus awkeotsang* Makino) achenes (JFA) and their acid hydrolysates by the ninhydrin test.

Tested Substances	Absorbance at 570 nm	
	Blank	Acid Hydrolysis
Gelatin	0.36 ± 0.03	9.10 ± 0.45
100% MeOHfr	0.03 ± 0.01	0.03 ± 0.02

Data are mean ± SD from three independent experiments. 100% MeOHfr, 100% methanol fraction of acetone soluble fraction from HP20 chromatography. S_PEI_, substance with pectin methylesterase inhibitory activity.

**Table 2:** Effect of JFA-S_PEI_ fractions on α-amylase and trypsin catalysis activity.

Treatments	α-Amylase Relative Activity (%)	Trypsin Relative Activity (%)
Control without S_PEI_	100 ^a^	100 ^a^
ASfr	76.8 ± 7.8 ^b^	88.6 ± 0.3 ^b^
100% MeOHfr	78.4 ± 11.5 ^b^	70.2 ± 4.2 ^c^

Data are mean ± SD from three independent experiments. Different letters are significantly different (*p* < 0.05) by Duncan’s multiple comparisons. Relative activity is the percentage of UV absorbance of tested groups to the control. S_PEI_, substances with pectin methylesterase inhibitory activity. ASfr, 90% acetone soluble fraction of S_PEI_ from jelly fig achenes. MeOHfr, methanol fraction of ASfr from HP20 chromatography.

**Table 3:** Tannin content of each isolated fraction from JFA.

Fractions	Tannins	
	Content in Tested Fractions (TAE mg/mL)	Total Tannins Content Divided by Dry Weight of Raw Materials (TAE mg/g D.W.)
Crude extract	8.48 ± 1.79 ^a^	1.70
ASfr	7.05 ± 1.21 ^ab^	1.41
100% water fraction	0.12 ± 0.04 ^e^	0.02
30% MeOHfr	0.16 ± 0.09 ^e^	0.03
100% MeOHfr	6.74 ± 1.93 ^b^	1.35
APfr	0.57 ± 0.11 ^c^	0.12
ASfr acid hydrolysate	0.32 ± 0.25 ^cd^	0.06
100% MeOHfr acid hydrolysate	0.22 ± 0.16 ^d^	0.04

Data are mean ± SD from three independent experiments. Different letters are significantly different (*p* < 0.05) by Duncan’s multiple comparisons. JFA, jelly fig achenes. TAE, tannic acid equivalent. ASfr, 90% acetone soluble fraction of S_PEI_ from jelly fig achenes. S_PEI_, substances with pectin methylesterase inhibitory activity. MeOHfr, methanol fraction of ASfr from HP20 chromatography.

## Supplementary Materials

The following are available online, Figure S1: Chromatographic profiles and appearance of acid hydrolysis resultant (1 N HCl, 121 °C for 30 min) of SPEI’s acetone-soluble fraction (ASfr). SPEI, substances with pectin methylesterase inhibitory activity.
