# Supplementary material for: Complex Tannins Isolated from Jelly Fig Achenes Affect Pectin Gelation through Non-Specific Inhibitory Effect on Pectin Methylesterase
Source: Molecules. 2019 Apr 23;24(8):1601. doi: 10.3390/molecules24081601 (PMC6515263; doi:10.3390/molecules24081601)
Supplement: Supplementary file 1 [file molecules-24-01601-s001.pdf]

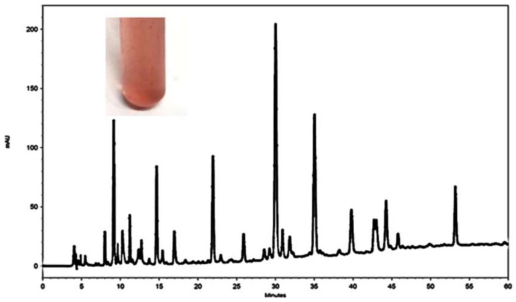

Figure S1. Chromatographic profiles and appearance of acid hydrolysis resultant (1 N HCl, 121 °C for 30 min) of S_{PEI}'s acetone-soluble fraction (ASfr). S_{PEI}, substances with pectin methylesterase inhibitory activity.
